# Can Pyomelanin Produced by *Pseudomonas aeruginosa* Promote the Regeneration of Gastric Epithelial Cells and Enhance *Helicobacter pylori* Phagocytosis?

**DOI:** 10.3390/ijms241813911

**Published:** 2023-09-10

**Authors:** Mateusz M. Urbaniak, Karolina Rudnicka, Grażyna Gościniak, Magdalena Chmiela

**Affiliations:** 1 Department of Immunology and Infectious Biology, Faculty of Biology and Environmental Protection, University of Łódź, 90-237 Łódź, Poland; karolina.rudnicka@biol.uni.lodz.pl; 2 Bio-Med-Chem Doctoral School, University of Lodz and Lodz Institutes of the Polish Academy of Sciences, 90-237 Łódź, Poland; 3 Department of Microbiology, Faculty of Medicine, Wrocław Medical University, 50-368 Wrocław, Poland; grazyna.gosciniak@umed.wroc.pl

**Keywords:** pyomelanin, *Helicobacter pylori*, immunomodulation, apoptosis, phagocytosis

## Abstract

*Helicobacter pylori* (*H. pylori*) infection is the most common cause of chronic gastritis, peptic ulcers and gastric cancer. Successful colonization of the stomach by *H. pylori* is related to the complex interactions of these bacteria and its components with host cells. The growing antibiotic resistance of *H. pylori* and various mechanisms of evading the immune response have forced the search for new biologically active substances that exhibit antibacterial properties and limit the harmful effects of these bacteria on gastric epithelial cells and immune cells. In this study, the usefulness of pyomelanin (PyoM) produced by *Pseudomonas aeruginosa* for inhibiting the metabolic activity of *H. pylori* was evaluated using the resazurin reduction assay, as well as in vitro cell studies used to verify the cytoprotective, anti-apoptotic and pro-regenerative effects of PyoM in the *H. pylori* LPS environment. We have shown that both water-soluble (PyoM_sol_) and water-insoluble (PyoM_insol_) PyoM exhibit similar antibacterial properties against selected reference and clinical strains of *H. pylori*. This study showed that PyoM at a 1 μg/mL concentration reduced *H. pylori*-driven apoptosis and reactive oxygen species (ROS) production in fibroblasts, monocytes or gastric epithelial cells. In addition, PyoM enhanced the phagocytosis of *H. pylori*. PyoM_sol_ showed better pro-regenerative and immunomodulatory activities than PyoM_insol_.

## 1. Introduction

*Helicobacter pylori* (*H. pylori*) is a Gram-negative, helix-shaped, microaerophilic bacterium that colonizes the gastric mucosa of approximately half of the world’s population [1]. *H. pylori* is one of the most successful gastric pathogens causing gastric and duodenal ulcers, gastric adenocarcinoma, mucosa-associated lymphoid tissue (MALT) lymphoma, and potentially other health dysfunctions leading to chronic inflammation, including coronary artery disease, iron deficiency anemia and autoimmune diseases [2,3,4]. The co-evolution of *H. pylori* within the human host has led to the development of efficient mechanisms for evading the innate immune response and the ability to colonize a gastric niche [5].

The primary adaptation mechanism of *H. pylori* is urease production, resulting in the alkalization of the acidic environment in the stomach and micro-niche formation ideal for *H. pylori* colonization followed by disruption of the intracellular tight junctions between epithelial cells and induction of an inflammatory response. Urease downregulates the production of arginase II and bactericidal nitric oxide (NO) in macrophages and promotes the survival of *H. pylori* within megasomes [6,7,8,9]. Other *H. pylori* virulence factors responsible for the disintegration of the gastric mucosa include vacuolating cytotoxin A (VacA) and cytotoxin-associated gene A (CagA) protein [10]. VacA inhibits the lysosomal and autophagic killing of *H. pylori*, establishing an intracellular niche for bacterial survival in macrophages [11]. CagA is an oncoprotein that mediates a range of intra-cellular effects in gastric epithelial cells by affecting several signalling pathways to promote chronic inflammation and proliferation of gastric epithelial cells that change their polarity and morphology, contributing to an increased risk of gastritis or neoplasia [12,13].

*H. pylori* and components of these bacteria, including lipopolysaccharide (LPS), induce apoptosis of gastric epithelial cells and macrophages [14,15,16]. Furthermore, *H. pylori* LPS mimics the carbohydrate structures—Lewis antigens present in human gastric mucosa, erythrocytes, and endothelium, which may weaken the host’s immune response towards these bacteria [17,18]. However, Lewis determinants of *H. pylori* LPS may drive the production of antibodies cross-reacting with the host components, resulting in the elevation of the complement-dependent inflammatory response [19]. Prolonged exposure of gastric epithelial cells to high levels of reactive oxygen species (ROS) generated during *H. pylori-induced* inflammation and subsequent redox imbalance lead to DNA damage, impairment of DNA repair mechanisms and cell apoptosis/necrosis [20].

The ability of *H. pylori* to avoid the response of innate immune mechanisms and the constant increase in antibiotic resistance are significant challenges in treating *H. pylori* infections [21]. A meta-analysis of 120 studies evaluating the effectiveness of first-line anti-*H. pylori* therapy showed that the eradication rate achieved in patients infected with resistant strains was only 67.4% [22]. The development of new drugs capable of eradicating *H. pylori* is highly recommended. One of the recent strategies concentrates on using bacterial metabolites, which may contribute to *H. pylori* eradication and modulation of the effector immune mechanisms impaired during infection. The potential candidate is pyomelanin (PyoM), a black-brown negatively charged extracellular polymer of homogentisic acid produced during L-tyrosine catabolism by *Pseudomonas aeruginosa* [23,24].

The presence of quinone structures in the PyoM molecule determines its oxidizing and reducing properties that allow for controlling the level of ROS [25]. The primary function of PyoM is protecting the bacterial cell from UV radiation [26] and the extracellular transfer of electrons [27]. The use of PyoM in the treatment of infections associated with excessive oxidative stress generated during inflammation is being considered [28]. Increased sensitivity of several bacterial pathogens to the antibiotics in the milieu of PyoM has been revealed, as well as the antibacterial activity of melanin against *H. pylori* has been demonstrated [29,30].

The study aimed to characterize the biological effects of water-soluble (PyoM_sol_) and water-insoluble pyomelanin (PyoM_insol_) regarding cytoprotective and pro-regenerative activity towards gastric epithelial cells. In addition, the antibacterial properties against *H. pylori* and the phagocytic efficiency of PyoM-stimulated monocytes, limited by the *H. pylori* component, were assessed.

## 2. Results

### 2.1. Antibacterial Activity of PyoM towards H. pylori

**Figure 1:**
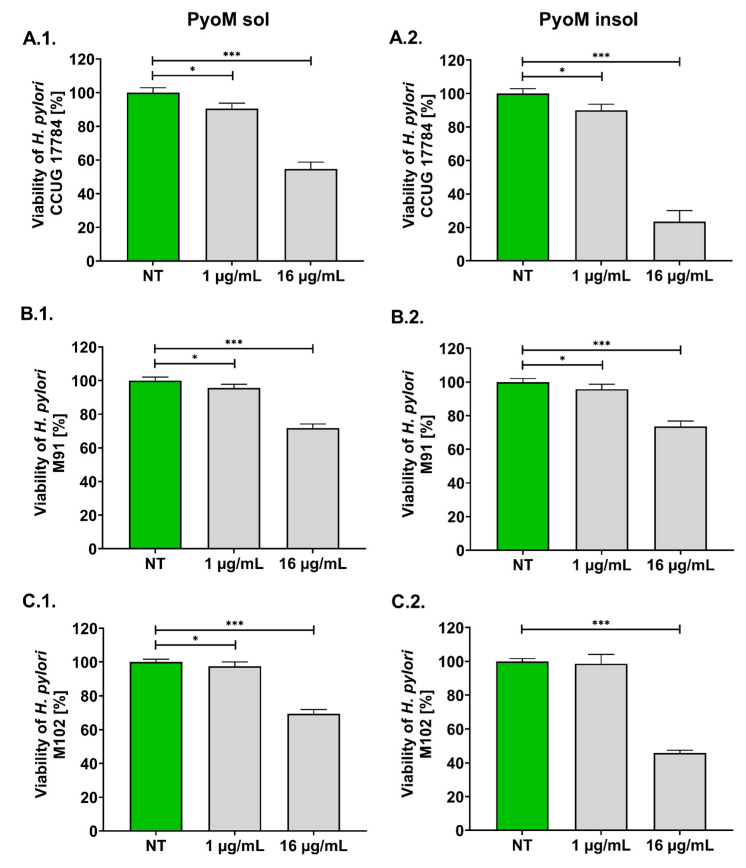
Antibacterial activity of pyomelanin against *Helicobacter pylori* strains. (**1**) Water-soluble pyomelanin (PyoM_sol_) and (**2**) water-insoluble pyomelanin (PyoM_insol_) were used at a concentration of 1 µg/mL or 16 µg/mL. *H. pylori* strains: (**A**) CCUG 17784, (**B**) M91 and (**C**) M102. Results are shown as means with standard deviations (SD) of four independent experiments performed in four replicates for each experimental variant. Statistical significance for * *p* < 0.05; *** *p* < 0.001; NT—untreated bacterial cells.

**Figure 2:**
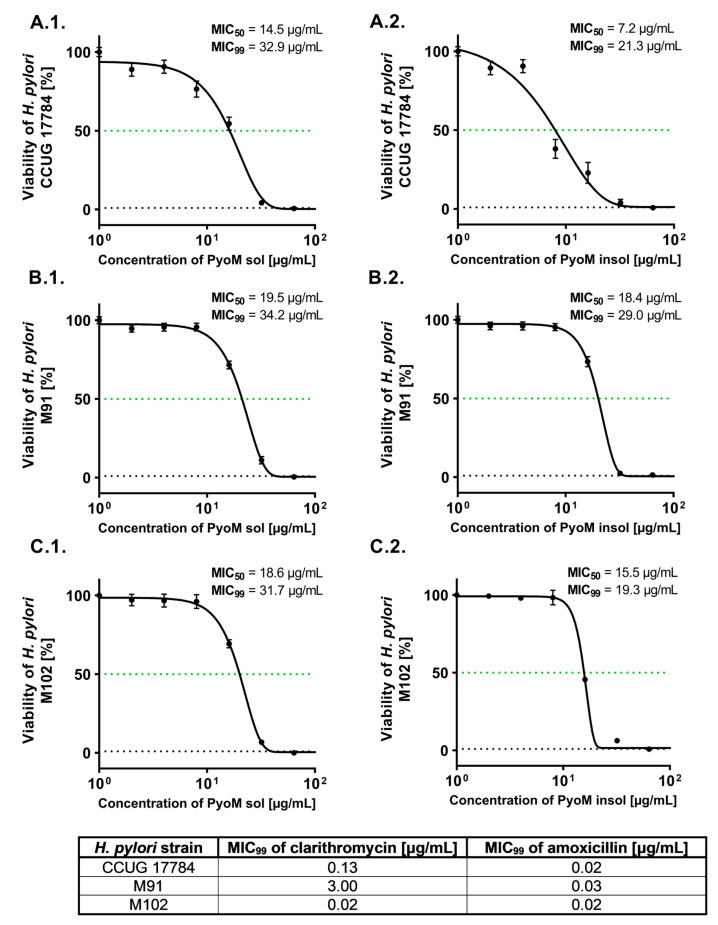
Dose–response curves and minimum inhibitory concentration (MIC)_50_ and MIC_99_ of pyomelanin and reference antibiotics against *Helicobacter pylori* strains, clarithromycin or amoxicillin. (**1**) Water-soluble (PyoM_sol_) and (**2**) water-insoluble pyomelanin (PyoM_insol_). *H. pylori* strains: (**A**) CCUG 17784, (**B**) M91 and (**C**) M102. Results are shown as means with standard deviations (SD) of four independent experiments performed in four replicates for each experimental variant. The green line indicates the 50% level of *H. pylori* viability.

Both forms of PyoM, PyoM_insol_ and PyoM_sol_, significantly reduced the viability of reference and clinical strains of *H. pylori* as examined by the resazurin reduction assay (Figure 1). The percentage of viable bacteria treated with PyoM formulations at a concentration of 16 μg/mL (*p* < 0.001) was significantly lower than the percentage of viable bacilli exposed to PyoM at a concentration of 1 μg/mL. Dose–response curves and MIC_50_ as well as MIC_99_ of PyoM against the reference *H. pylori* CCUG 17784 and two clinical isolates, *H. pylori* M91 and *H. pylori* M102 are shown in Figure 2. MIC_50_ and MIC_99_ were defined as the lowest concentration of the PyoM at which 50% and 99% of the *H. pylori* cells were killed, respectively. The PyoM_sol_ MIC_50_ towards the reference *H. pylori* CCUG 17784 strain was 14.5 µg/mL and 19.5 µg/mL for *H. pylori* M91 or 18.6 µg/mL for *H. pylori* M102. The PyoM_insol_ MIC_50_ against *H. pylori* CCUG 17784 was 7.2 μg/mL, while for *H. pylori* M91 was 18.4 μg/mL and 15.5 μg/mL towards *H. pylori* M102. The MIC_99_ of PyoM_sol_ against the studied *H. pylori* strains was in the range 31.7–34.2 μg/mL, while MIC_99_ of PyoM_insol_ was in the range 19.3–29.0 μg/mL. For all *H. pylori* strains, the MIC_99_ of clarithromycin or amoxicillin was significantly (*p* < 0.001) lower compared to the MIC of both forms of PyoM (Figure 2).

### 2.2. PyoM Neutralizes the Cytotoxic Effect of H. pylori LPS towards Gastric Epithelial Cells and Monocytes

**Figure 3:**
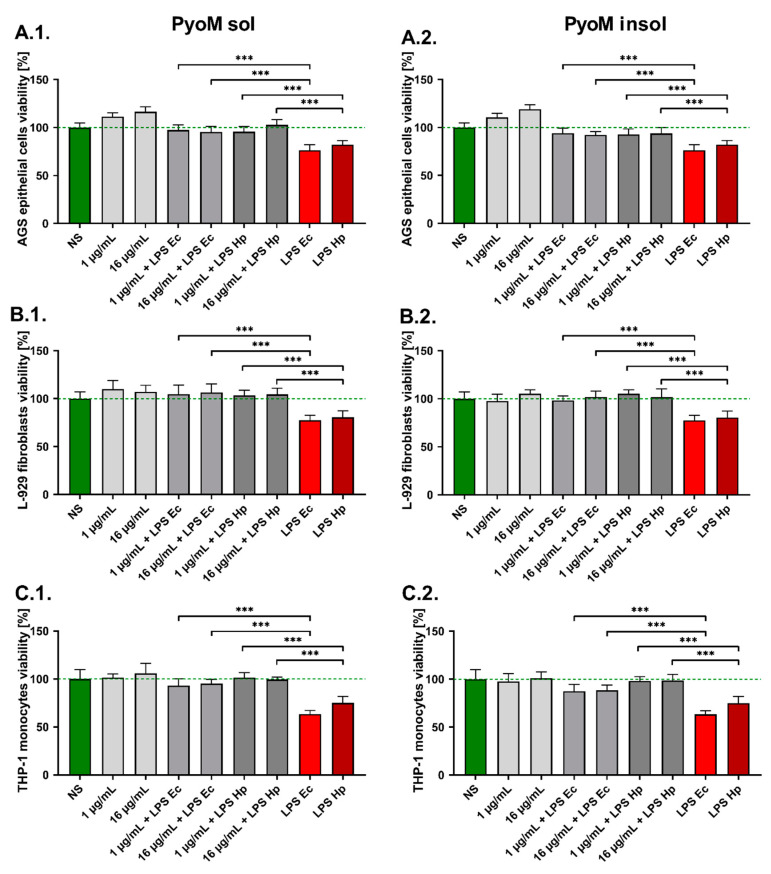
Reversion of the cytotoxic effect of *H. pylori* lipopolysaccharide (LPS) or *E. coli* LPS by pyomelanin. (**1**) Water-soluble (PyoM_sol_) or (**2**) water-insoluble pyomelanin (PyoM_insol_) at a concentration of 1 µg/mL or 16 µg/mL. Cell cultures: (**A**) AGS gastric epithelial cells, (**B**) L-929 mouse fibroblasts and (**C**) THP-1 human monocytes. Cells were stimulated for 24 h with PyoM alone, lipopolysaccharide (LPS) *H. pylori* (LPS Hp) or LPS *E. coli* (LPS Ec) alone or both. Results are shown as means with standard deviations (SD) of six independent experiments performed in six replicates for each experimental variant. Statistical significance for *** *p* < 0.001. NS—unstimulated cells. The green line indicates the reference viability of unstimulated cells.

We showed that PyoM_sol_ and PyoM_insol_ did not affect the metabolic activity and thus the viability of reference mouse fibroblasts L-929, human gastric epithelial cells AGS and human THP-1 monocytes at the concentration of 1 µg/mL or 16 µg/mL (Figure 3). In cell cultures exposed for 24 h to *H. pylori* LPS alone, the percentage of viable cells significantly (*p* < 0.001) decreased to 75.1% ± 6.8%, 82.2% ± 4.3% and 80.5% ± 6.8%, respectively. Similarly, the viability of studied cells in the presence of LPS *E. coli* significantly (*p* < 0.001) diminished. In cell cultures treated simultaneously with PyoM_sol_ or PyoM_insol_ at a concentration of 1 μg/mL or 16 μg/mL and *H. pylori* or *E. coli* LPS, the cell viability was similar to the viability of untreated cells, which suggests that PyoM neutralized the cytotoxic effect of LPS (*p* < 0.001).

### 2.3. LPS-Induced Apoptosis Is Diminished in the Presence of PyoM_sol_

The PyoM_sol_-mediated neutralization of *H. pylori* LPS-induced cytotoxicity prompted us to examine whether the inhibition of cell apoptosis accompanies this phenomenon.

**Figure 4:**
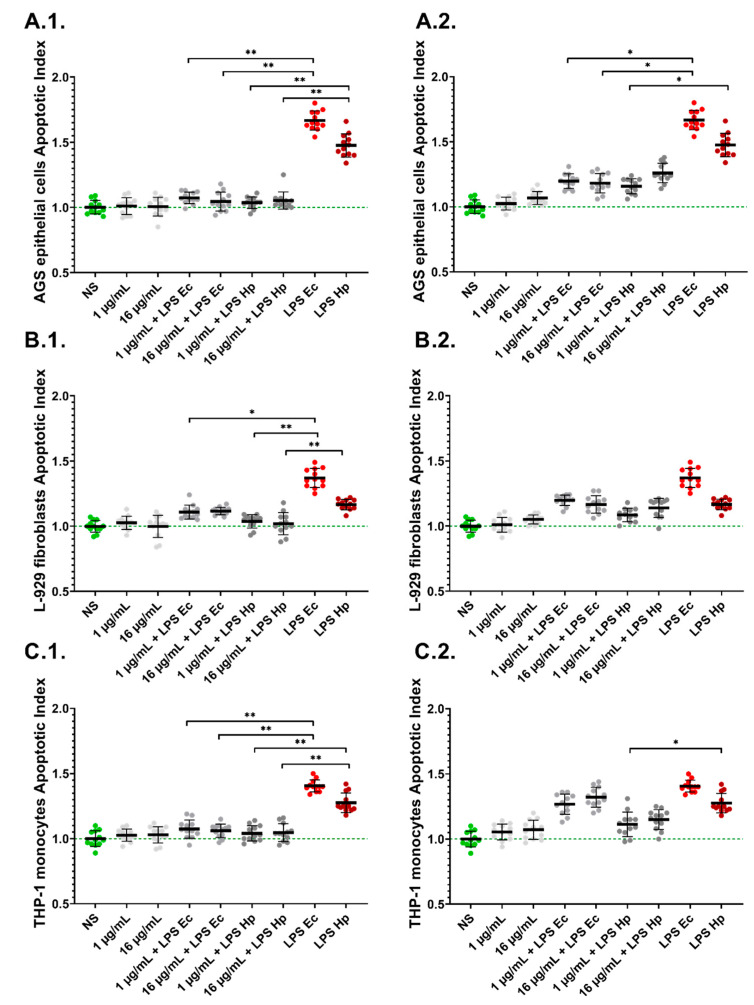
Diminishing cell apoptosis induced by *H. pylori* lipopolysaccharide (LPS) or *E.coli* LPS in the milieu of pyomelanin in cell cultures in vitro. (**1**) Water-soluble pyomelanin (PyoM_sol_) or (**2**) water-insoluble pyomelanin (PyoM_insol_) were used at a concentration of 1 µg/mL or 16 µg/mL. (**A**) AGS gastric epithelial cells, (**B**) L-929 mouse fibroblasts and (**C**) THP-1 human monocytes were stimulated for 24 h with PyoM alone, lipopolysaccharide (LPS) *H. pylori* (LPS Hp) or LPS *E. coli* (LPS Ec) alone or both. The Apoptotic Index was calculated based on the relative fluorescence units (RFU) of stimulated cells vs. RFU of the control unstimulated cells. Results are shown as means with standard deviations (SD) of four independent experiments performed in triplicate for each experimental variant. Statistical significance for * *p* < 0.05; ** *p* < 0.01. NS—unstimulated cells. The green line indicates the reference Apoptotic Index of unstimulated cells.

Neither PyoM_sol_ nor PyoM_insol_ induced cell apoptosis manifested by the lack of an increase in the Apoptotic Index (AI) compared to untreated cells (Figure 4). The AI in AGS, L-929 and THP-1 cells increased significantly (*p* < 0.001) after cell exposure to *H. pylori* LPS compared to untreated cells (AI = 1.00), and was equal to 1.48 ± 0.09, 1.17 ± 0.04 and 1.28 ± 0.08, respectively. *E. coli* LPS, similar to *H. pylori* LPS, significantly (*p* < 0.001) increased the AI of gastric epithelial cells, fibroblasts and monocytes. In cell cultures carried out in the presence of PyoM_sol_, the cell apoptosis induced by *H. pylori* LPS or *E.coli* LPS significantly diminished (*p* < 0.01) compared to the level of apoptosis after cell stimulation with *H. pylori* or *E. coli* LPS alone. However, PyoM_insol_ only at a concentration of 1 μg/mL (*p* < 0.05) decreased the AI of AGS and THP-1 cells co-stimulated with *H. pylori* LPS. By comparison, for PyoM_insol_ (16 μg/mL), a reduction in the AI was demonstrated only for AGS cells co-stimulated with *E. coli* LPS.

### 2.4. PyoM Neutralizes Reactive Oxygen Species Produced by Cells Exposed to H. pylori LPS

**Figure 5:**
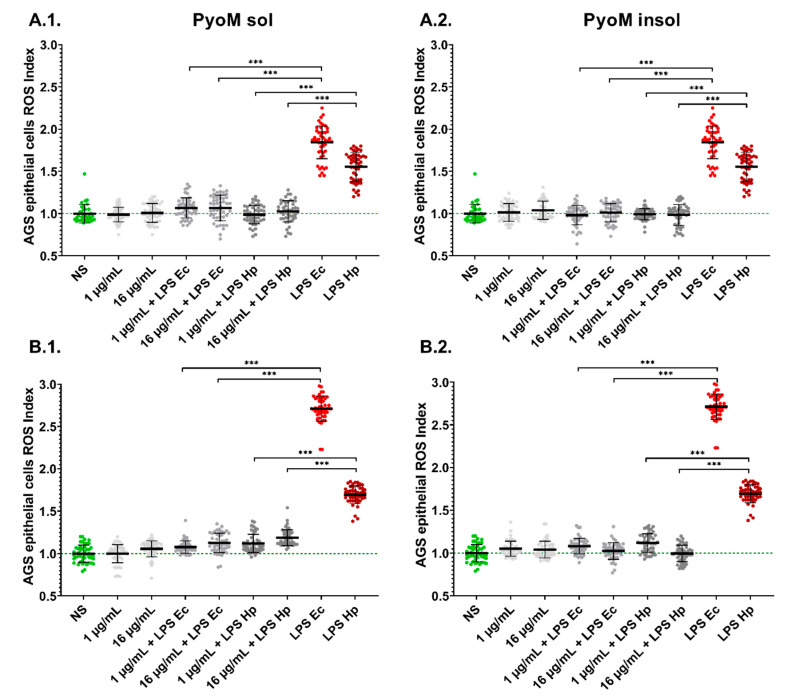
Neutralization by pyomelanin of reactive oxygen species (ROS) produced by AGS gastric epithelial cells. Cells were stimulated for 24 h with (**1**) water-soluble pyomelanin (PyoM_sol_) or (**2**) water-insoluble pyomelanin (PyoM_insol_) at a concentration of 1 µg/mL or 16µg/mL alone or with lipopolysaccharide (LPS) of *H. pylori* (LPS Hp) or *E. coli* LPS (LPS Ec) in the milieu with or without pyomelanin (PyoM). ROS production index after (**A**) 30 min or (**B**) 24 h of cell stimulation with (**1**) PyoM_sol_ or (**2**) PyoM_insol_. The ROS Index was calculated based on the relative fluorescence units (RFU) of stimulated cells vs. RFU of control unstimulated cells. Results are shown as means with standard deviations (SD) of six independent experiments performed in eight replicates for each experimental variant. Statistical significance for *** *p* < 0.001. NS—unstimulated cells. The green line marks the reference ROS Index of unstimulated cells.

The LPS of *H. pylori* and the reference *E. coli* LPS induced significantly higher (*p* < 0.001) ROS production in AGS cells after 30 min or 24 h as compared to the unstimulated cells (Figure 5). The AGS gastric epithelial cells after long-term (24 h) exposure to *H. pylori* LPS responded by ROS production more effectively than these cells after short-term (30 min) stimulation. Both forms of PyoM induced a significant (*p* < 0.001) reduction of ROS in cell cultures co-stimulated with PyoM and *H. pylori* LPS or *E. coli* LPS when compared to cells stimulated with LPS alone.

### 2.5. PyoM_sol_ Promotes the Migration of Gastric Epithelial Cells Affected by H. pylori LPS

**Figure 6:**
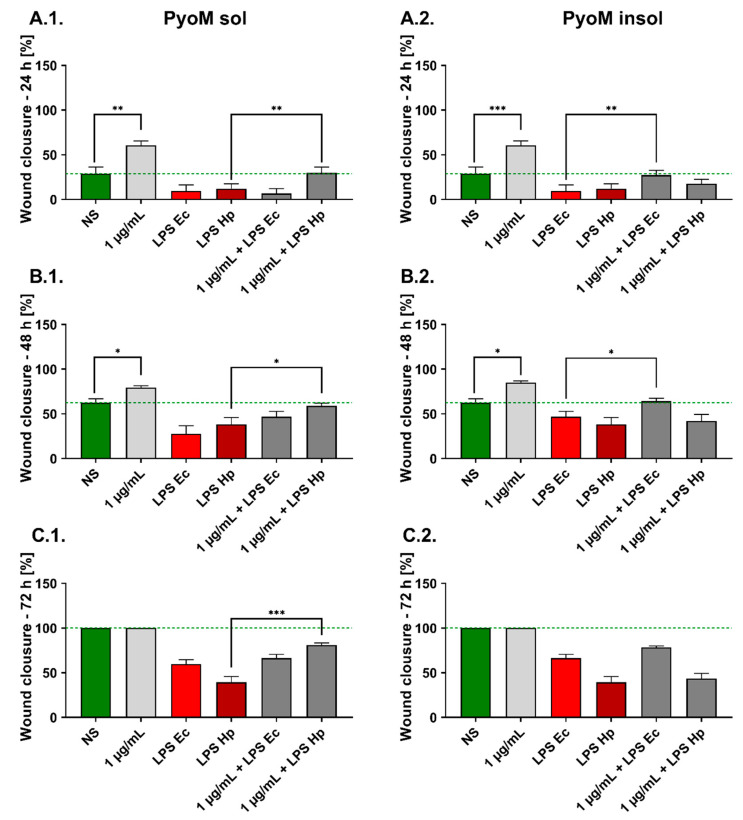
The effectiveness of AGS cell migration in wound healing assay. The cell cultures were exposed to (**1**) water-soluble pyomelanin (PyoM_sol_) or (**2**) water-insoluble pyomelanin (PyoM_insol_) alone, lipopolysaccharide (LPS) of *H. pylori* (LPS Hp) or *E.coli* LPS (LPS Ec) alone or both to pyomelanin and LPS for (**A**) 24 h, (**B**) 48 h or (**C**) 72 h. Results are shown as means with standard deviations (SD) of four independent experiments performed in triplicate for each experimental variant. Statistical significance for * *p* < 0.05; ** *p* < 0.01; *** *p* < 0.001. NS—unstimulated cells. The green line indicates the reference wound clousure of unstimulated cells.

The migration of untreated AGS cells increased with time and the average percentages of cells in the wounded zone were: 28.9% ± 7.6%, 62.5% ± 4.5% and 100% after 24, 48 and 72 h, respectively (Figure 6). PyoM_sol_ and PyoM_insol_ significantly stimulated the cell migration within the wound after 24 and 48 h, and the wound healing rate was 60.7% ± 4.8% and 85.2% ± 1.8% for PyoM_sol_ and 60.5 ± 4.9% and 79.3% ± 2.2% for PyoM_insol_, respectively. *E. coli* LPS significantly affected the cell migration by up to 9.7% ± 6.6%, 46.8% ± 6.0%, and 66.3% ± 4.3% confluence in 24, 48 and 72 h cell cultures, respectively. *H. pylori* LPS at the same time points significantly reduced the rate of AGS cell migration to 12.2% ± 5.3%, 38.2% ± 7.8% and 39.4% ± 6.3%, respectively. Co-stimulation of AGS cells with PyoM_sol_ and *H. pylori* LPS resulted in an increased wound closure to 29.7% ± 6.6%, 59.0% ± 3.1% and 81.0% ± 2.4% after 24, 48, and 72 h, respectively, when compared to the effect induced by LPS used separately. Compared to the effect of LPS used individually, co-stimulation of AGS cells with PyoM_insol_ and *E. coli* LPS increased the rate of wound closure by 27.2% ± 5.5% and 64.2% ± 3.2% after 24 and 48 h, respectively. We have shown that co-stimulation of AGS cells with PyoM_sol_ and *E. coli* LPS did not influence the rate of wound closure compared to the cells treated with LPS alone. Similar results were shown in AGS cell cultures exposed to PyoM_insol_ and *H. pylori* LPS.

### 2.6. PyoM_sol_ Enhances the Phagocytic Capacity of Monocytes towards Fluorescently Labelled E. coli or H. pylori

**Figure 7:**
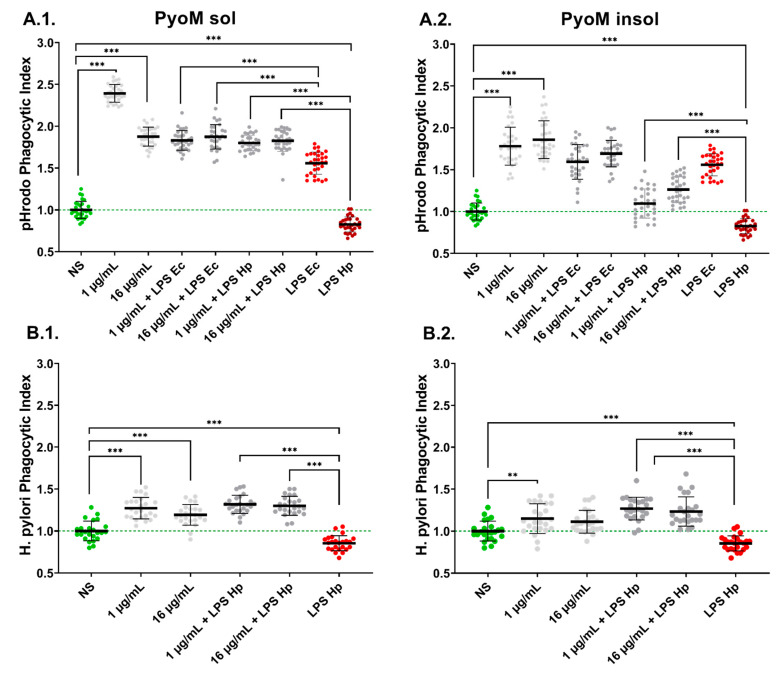
Upregulation of phagocytic activity of THP-1 human monocytes towards a fluorescently labelled *E.coli* or *H. pylori*, in vitro. THP-1 cells were stimulated with (**1**) water-soluble (PyoM_sol_) or (**2**) water-insoluble pyomelanin (PyoM_insol_), lipopolysaccharide (LPS) of *H. pylori* (LPS Hp) or *E. coli* LPS (LPS Ec) alone or co-stimulated with the studied formulations of PyoM and *H. pylori* LPS or *E.coli* LPS. (**A**) Phagocytosis of reference *E. coli* pHrodo™ and (**B**) phagocytosis of *H. pylori* fluorescently labelled *H. pylori* with BacLight™. The Phagocytic Index was calculated based on relative fluorescence units (RFU) of stimulated cells vs. RFU of control unstimulated cells. Results are shown as means with standard deviations (SD) of four experiments performed in six replicates for each experimental variant. Statistical significance for ** *p* < 0.01; *** *p* < 0.001. NS—unstimulated cells. The green line indicates the reference Phagocytic Index of unstimulated cells.

The fluorescently labelled reference *E. coli* (*E. coli* pHrodo™) or *H. pylori* were used in the phagocytosis assay performed with THP-1 monocytes exposed for 24 h to *H. pylori* LPS or *E. coli* LPS alone, PyoM alone or to LPS and PyoM. The results of the phagocytosis assay expressed as a Phagocytic Index (PI) are shown in Figure 7. The stimulation of monocytes with PyoM_sol_ or PyoM_insol_ resulted in a significant (*p* < 0.001) improvement in the phagocytosis of the reference *E. coli* pHrodo™. By comparison, *H. pylori* LPS significantly (*p* < 0.001) reduced the ability of monocytes to engulf *E. coli* pHrodo™ compared to untreated cells. However, co-stimulation of THP-1 monocytes with PyoM_sol_ (1 and 16 μg/mL) or PyoM_insol_ (1 μg/mL) and *H. pylori* LPS resulted in a reversion of the LPS-induced inhibition of phagocytosis. In addition, PyoM_sol_, but not PyoM_insol_, induced a significant (*p* < 0.001) increase in the phagocytic index of THP-1 monocytes co-stimulated with *E. coli* LPS, compared to those cells treated solely with LPS alone. In the case of live fluorescently labelled *H. pylori*, the PyoM_sol_ at a concentration of 1 μg/mL or 16 μg/mL significantly (*p* < 0.001) increased the PI of THP-1 monocytes compared to untreated cells. PyoM_insol_ showed a similar effect, however, only at a concentration of 1 μg/mL.

## 3. Discussion

The discovery of *H. pylori* in 1982 by Warren and Marshall proved that the stomach, with its acidic pH, can be colonized by these bacteria [5]. Development of gastritis, gastric or duodenal ulcers, and even gastric cancer due to *H. pylori* infection depends on bacterial virulence factors, susceptibility of the host, efficiency of the immune mechanisms, and environmental conditions [31]. The increasing antibiotic resistance of this pathogen requires the search for new therapeutic agents with antibacterial activity [32,33]. Moreover, cytoprotective and immunomodulatory activity of therapeutic formulations should also be considered. This study demonstrated that PyoM_sol_ and PyoM_insol_ exhibit antibacterial activity against the reference and clinical *H. pylori* strains. The antibacterial activity of bacterial melanins was previously described by Vasanthabharathi et al., who showed that a pigment isolated from a marine strain of *Streptomyces* sp. inhibited the growth of *E. coli* and *Lactobacillus vulgaris* [34]. Zerrad et al. reported that melanin isolated from *Pseudomonas balearica* showed strong antimicrobial activity against *Staphylococcus aureus*, *E. coli* and *Candida albicans*, as well as the phytopathogenic *Erwinia chrysanthemi* and *E. carotovora* [35]. Xu et al. have suggested that the toxicity of fungal melanin towards *Vibrio parahaemolyticus* and *S. aureus* is related to the impairment in the bacterial cell membrane of these pathogens [36]. It was shown that melanin and glucan complexes diminish the viability of *C. albicans* and *H. pylori* [37].

Melanin compounds are known to bind redox active metal ions, including iron ions (Fe^2+^/Fe^3+^) [38]. The study of Waidner et al. showed that disturbances in the uptake of Fe ions by *H. pylori* result in reduced bacterial activity and the ability to colonize the stomach [39]. PyoM, by reduction of soluble Fe to insoluble Fe, provides homeostasis for Fe^2+^/Fe^3+^ ions, which is necessary for the survival of pyomelaninogenic bacteria [40]. Reducing the solubility of Fe in the *H. pylori* niche may result in disturbances in ion metabolism and limit the viability of these bacteria. Further studies are needed to confirm the iron-dependent mechanism of PyoM antibacterial activity against *H. pylori.*

We have shown that PyoM has a cytoprotective effect on fibroblasts, gastric epithelial cells and monocytes, which were primed with *H. pylori* LPS or the reference *E. coli* LPS in vitro. Potentially, in vivo PyoM may protect the gastric epithelium from *H. pylori*-driven damage and prevent the infiltration of *H. pylori* components through the epithelial barrier. These *H. pylori*-related effects have been demonstrated in earlier studies [16,41,42].

In this study, we determined the level of cell viability, apoptosis and ROS production to verify the cytoprotective mechanism of PyoM against *H. pylori* LPS-driven deleterious effects.

Apoptosis plays an essential role in gastric tissue physiology [43]. Especially, in *H. pylori*-induced chronic gastritis, where excessive apoptotic cell loss dominates over cell proliferation [44]. It has been shown that *H. pylori* and soluble components of these bacteria, including LPS, increase ROS generation in the gastric mucosa, which may contribute to apoptosis of gastric epithelial cells. In a model of primary gastric epithelial cells and fibroblasts of guinea pig, it was revealed that *H. pylori* LPS-mediated upregulation of ROS leads to an increased rate of apoptosis and reduced cell-to-cell integrity [16,45]. In this study, the percentage of cells undergoing apoptosis in the milieu of *H. pylori* LPS, in cell cultures of gastric epithelial cells, fibroblasts or monocytes in vitro, has been diminished in the presence of PyoM_sol_. Further in vivo studies are needed to confirm this effect.

In *H. pylori*-infected patients, the increased ROS can be delivered by granulocytes and macrophages recruited to the gastric mucosa in response to chemotactic signals. Ding et al. showed that *H. pylori* may also generate ROS directly and contribute to the accumulation of ROS in gastric tissues [46]. *H. pylori* infection also contributes to the production of reactive nitrogen species (RNS), and this effect is related to nicotinamide adenine dinucleotide phosphate oxidase (Nox) and inducible nitric oxide synthase (iNOS) production [14]. Moreover, neutrophils and gastric epithelial cells also express iNOS, involved in the production of NO, which reacts with metal ions and ion superoxide, producing a strong oxidant—peroxynitrite [14,47]. Patients infected with *H. pylori* have elevated levels of ROS along with increased activity of NO-derived metabolites, indicating the activation of iNOS [48].

Lorguin et al., using a human keratinocyte model, showed that PyoM effectively suppresses ROS activity in cells exposed to UVA light [26]. Our studies have shown that both PyoM_sol_ and PyoM_insol_ neutralized the short-term and long-term activity of intracellular ROS induced by *H. pylori* LPS in a model of gastric epithelial cells, which was correlated with diminishing the number of cells undergoing apoptosis. These cytoprotective properties of PyoM are very promising since the leakage of the gastric epithelial barrier due to uncontrolled ROS production and excessive apoptosis may facilitate the systemic distribution of *H. pylori* components [41]. In the present study, we showed that PyoM_sol_ selectively improved the cell migration affected by *H. pylori* LPS, while PyoM_insol_ upregulated the cell migration inhibited in the presence of *E. coli* LPS. A different profile of cell migration in cell cultures exposed to *H. pylori* LPS or *E. coli* LPS and PyoM may result from some differences in the chemical structure of PyoM_sol_ and PyoM_insol_, as well as their ability to bind individual bacterial LPS, *H. pylori* LPS or *E. coli* LPS.

Professional phagocytes remove pathogenic microorganisms and apoptotic cells and initiate an adaptive immune response by presenting antigens to lymphocytes [49]. It has been shown that in the presence of *H. pylori* LPS, the engulfment of these bacteria by phagocytes is diminished [50,51,52]. Also, surface haemagglutinins of *H. pylori* and urease may downregulate the ability of phagocytes to ingest these bacteria [6,53]. We have shown that PyoM_sol_ and PyoM_insol_ at a concentration of 1 μg/mL or 16 μg/mL effectively stimulated monocytes to increase phagocytosis of the reference *E. coli* pHrodo™. PyoM_sol_ in both concentrations enhanced the ingestion of fluorescently labelled *H. pylori*, while for PyoM_insol,_ only at 1 μg/mL. PyoM_sol_ also upregulated the engulfment of *E.coli* particles, which was diminished in the milieu of LPS *E. coli* or LPS *H. pylori*. In contrast, PyoM_insol_ only upregulated the phagocytic activity of monocytes diminished in the presence of *H. pylori* LPS; however, less effectively than PyoM_sol_. Moreover, PyoM_sol_ upregulated the ingestion of *H. pylori*, which was inhibited by *H. pylori* LPS. These results indicate that PyoM_sol_ possesses better immunomodulatory potential towards monocyte phagocytic activity than PyoM_insol_. Alviano et al. reported that soluble melanin isolated from *Fonsecaea pedrosoi* activated macrophages and neutrophils, resulting in increased phagocytosis and oxidative burst during incubation with *F. pedrosoi* and *C. albicans* [54].

*H. pylori* has developed a set of antioxidant proteins, including superoxide dismutase, catalase and arginase, which protect these bacteria from oxidative destruction during phagocytosis [55]. The question arises about the possible cellular mechanisms involved in upregulating *E. coli* or *H. pylori* engulfment by monocytes in the presence of PyoM, omitting antioxidative PyoM properties. PyoM possibly activates monocytes extracellularly through the cell surface receptors and induces metabolic reprogramming of phagocytes. A recent study by Chen et al. showed that fungal melanin of *Aspergillus fumigatus* may function as a pathogen-associated molecular pattern molecule. This fungal component induces in macrophages the activation of hypoxia-inducible factor 1 subunit alpha (HIF-1α) and phagosomal recruitment of mammalian target of rapamycin (mTOR)—Akt/mTOR/HIF1α axis resulting in a metabolic shift towards glycosylation, i.e., metabolic reprogramming of macrophages. As a result, the antimicrobial activity of macrophages was increased [56]. The study by Goncalves et al. revealed that by remodelling the calcium sequestration inside the phagosome and impairing signalling via calmodulin, fungal melanin drives the above glycosylation pathway [57]. In the case of gastric mucosa, the antioxidative properties of PyoM may improve the protection of epithelial cells and immune cells from deleterious oxidative stress generated in the *H. pylori*-driven inflammatory milieu. Protective properties of PyoM against monocyte death in the milieu of *H. pylori* components (including LPS) may result in more effective eradication of this pathogen.

In conclusion, PyoM seems to be a promising candidate for diminishing the negative effects caused by *H. pylori* in the gastric mucosa. The in vitro gastro-protective activity of PyoM is related to its anti-oxidative properties in conjunction with the control of cell apoptosis. PyoM also possesses pro-regenerative activity since it stimulates the cell migration affected by *H. pylori* LPS. We showed that the ability of monocytes to engulf fluorescently labelled *E. coli* or *H. pylori* is significantly enhanced in the presence of PyoM. From two studied PyoM formulations, PyoM_sol_ better stimulated the migration of gastric epithelial cells and phagocytic activity of monocytes, which were affected by LPS *H. pylori* than PyoM_insol_, and due to this, PyoM_sol_ is promising for further study in an in vivo model of *H. pylori* infection in *Caviae porcellus* (guinea pigs), which previously was characterized by us in terms of inflammatory and immune responses [52,58].

Despite the description of the antibacterial activity of PyoM, its bactericidal mechanism remains unknown, which is a limitation of this study. Further studies are required to determine the potential molecular targets for PyoM and the specificity of the antibacterial activity against Gram-negative and Gram-positive bacteria. Further research will be carried out to define the molecular basis underlying PyoM_sol_/PyoM_insol_-mediated cellular effects.

## 4. Materials and Methods

### 4.1. Growth Conditions of Pseudomonas aeruginosa

When starting a strain from a frozen stock, the *P. aeruginosa* Mel+ strain (Collection of Department of Immunology and Infectious Biology University of Łódź, Poland) was streaked out onto a Luria Broth (LB) agar plate, and a subsequent liquid culture started the next day. The LB broth was then inoculated with the single colony exhibiting Gram-negative rod-shaped morphology, positive oxidase test and a presence of pigmentation and incubated in aerobic conditions (37 °C, 18 h) to obtain an initial log-phase bacterial suspension. To isolate PyoM with the reduction of undesirable substances, the Pyomelanin Minimal Medium II (PMM II) (patent application number: PL438865) was used [24]. PMM II was inoculated with 1.0 mL of a 1.0 McFarland bacterial suspension and grown for five days in the microbiological incubator (37 °C, shaking at 120 rpm). When the colour of the medium changed from honey-like to black, the cultures were transferred to room temperature and exposed to sunlight, which stimulated the production of the pigment (48 h).

### 4.2. Isolation and Purification of PyoM_insol_ and PyoM_sol_

The isolation and purification of two variants of pyomelanin were performed as previously described [24]. To isolate the water-insoluble pyomelanin (PyoM_insol_), the bacterial culture was centrifuged (6600× *g*), and supernatant was acidified to pH 2.0 with 6.0 M HCl (PolAura, Dywity, Poland). The PyoM_insol_ pellet was washed with HCl and double-washed with distilled water. Finally, the Pyo_insol_ pellet was suspended in pure ethanol (Chempur, Piekary Śląskie, Poland), and placed in a water bath (95 °C, 30 min.). PyoM_insol_ was washed twice with ethanol and air-dried. In order to obtain water-soluble pyomelanin (PyoM_sol_), the bacterial cell-free supernatant was incubated with chloroform in a 1:1 ratio under shaking conditions for 24 h (room temperature, shaking at 120 rpm). The aqueous phase containing the PyoM_sol_ was separated from the chloroform and protein phase using a separating funnel. To remove residual protein contaminants, the aqueous layer was centrifuged (6600× *g*), and then PyoM_sol_ was purified from low molecular weight soluble substances by ultrafiltration (MWCO 5 kDa) (Sartorius, Göttingen, Germany). The PyoM_sol_ was dried overnight at 50 °C. Endotoxin was removed via affinity chromatography using Pierce™ High Capacity Endotoxin Removal Spin Columns (Thermo Scientific, Waltham, MA, USA). The resin and column were prepared and equilibrated according to the manufacturer’s protocol. The samples of PyoM_sol_ and PyoM_insol_ (5 mg/mL) were applied to the columns, incubated for 3 h with gentle mixing, centrifuged at 500× *g* and pellets were collected in the new tubes and dried at 50 °C overnight. PyoM_insol_ and PyoM_sol_ pellets were washed with chloroform, ethyl acetate, ethanol and water. For further experiments, the PyoM variants were stored in a dark and dry place at 4 °C.

### 4.3. Cell Cultures

The reference L-929 mouse fibroblasts, human AGS gastric adenocarcinoma epithelial cells and human THP-1 monocytes purchased from the American Type Culture Collection (ATCC, Rockville, MD, USA) were used and propagated as previously described [24,41]. Prior to experiments, L-929 and THP-1 cells were cultured in Roswell Park Memorial Institute (RPMI)-1640 medium supplemented with 10% heat-inactivated foetal calf serum (FCS; HyClone Cytiva, Marlborough, MA, USA), and the antibiotics penicillin (100 U/mL) and streptomycin (100 µg/mL) (Sigma-Aldrich, Darmstadt, Germany). AGS cells were grown in Dulbecco’s Modified Eagle Medium/Nutrient Mixture F-12 (DMEM/F-12; Biowest, Nuaillé, France), containing 10% heat inactivated FCS, and standard antibiotics. Cell cultures were incubated at 37 °C in a humidified atmosphere containing 5% CO_2_. The cells were passaged with 0.25% trypsin in 0.02% ethylenediaminetetraacetic acid (EDTA) (Biowest, Nuaillé, France). The cell viability and density were assessed using trypan blue (Blaubrand, Wertheim, Germany) exclusion assay. The cells were used in the experiments when cell’s viability was higher than 95%.

### 4.4. Cell Stimulators

The reference *Helicobacter pylori* strain CCUG 17874 (purchased from Culture Collection, University of Gothenburg, Gothenburg, Sweden), positive for vacuolating toxin A (VacA) and cytotoxin associated gene A (CagA) protein, was grown under microaerophilic conditions (76 h, 37 °C). LPS from the reference *H. pylori* strain was prepared by hot phenol–water extraction, purified by proteinase K and RNA-se treatment and ultracentrifugation as previously described [59]. *Escherichia coli* (*E. coli*) LPS O55:B5 (Sigma-Aldrich, Darmstadt, Germany) was used as positive control. The stock solution of PyoM_insol_ was prepared in 50 mM NaOH and then diluted in phosphate-buffered saline (PBS), pH 7.4. The detrimental effect of the solvent, diluted in PBS, has been excluded in the preliminary study as previously described [24]. The concentration of PyoM was equal to 1 and 16 μg/mL for PyoM_sol_ and PyoM_insol_, while the *H. pylori* LPS and *E.coli* LPS were used at a concentration of 25 ng/mL as described previously [16]. In cell-based assays, all components were dissolved in a cell culture medium dedicated to the appropriate cell line and sterilized by filtration using 0.22 μm pore size membrane filters (Sartorius, Göttingen, Germany).

### 4.5. Antibacterial Activity of PyoM_insol_ and PyoM_sol_

The antibacterial activity of PyoM_insol_ and PyoM_sol_ against the reference *H. pylori* CCUG 17874 and two clinical isolates, *H. pylori* M91 (metronidazole-resistant strain) and *H. pylori* M102 (metronidazole and levofloxacin-resistant strain), was determined as the minimum inhibitory concentration (MIC) by the resazurin reduction assay [60]. The bacteria were cultured in Brucella Broth with 10% FCS to mid-log phase, and the inoculum was adjusted to 0.5 McFarland (1.5 × 10^8^ CFU/mL) scale. Next, the bacterial suspension was diluted 100-fold in the medium. PyoM_insol_ and PyoM_sol_ were distributed into wells of a 96-well plate (Nunc, Rochester, NY, USA) containing 100 μL Brucella Broth with 10% FCS to form a series of 2-fold dilutions in the range of 1–1024 μg/mL. Then, the bacterial suspension (100 μL) was added to each well, and plates were incubated for 72 h at 37 °C. Control wells containing bacterial culture alone (positive control of bacterial growth), wells with bacterial medium alone (negative control) and wells with reference antibiotics (clarithromycin or amoxicillin) were included. To assess MIC, 20 μL of 0.02% resazurin in sterile PBS was added to each well and left for 3 h. Fluorescence was measured at an excitation wavelength of 560 nm and emission wavelength of 590 nm using a SpectraMax^®^ i3x Multi-Mode Microplate Reader (Molecular Devices, San Jose, CA, USA).

### 4.6. Cell’s Viability Assay

The viability of cells treated with PyoM, LPS or co-stimulated with PyoM and bacterial LPS was assessed using a 3-(4,5-dimethylthiazol-2-yl)-2,5-diphenyltetrazolium bromide—MTT (Sigma Aldrich, Darmstadt, Germany) reduction assay as previously described [24]. L-929 fibroblasts, AGS gastric adenocarcinoma epithelial cells or THP-1 monocytes adjusted to a density of 2 × 10^5^ cells/mL (100 μL) were seeded in 96-well culture plates (Nunc, Rochester, NY, USA) and incubated overnight prior to stimulation. Cell morphology and confluency were controlled using an inverted contrast phase microscope (Motic AE2000, Xiamen, China). The solution of tested PyoM was distributed to the wells of cell culture plates (6 replicates for each experimental variant) containing cell monolayers. After 24 h of incubation, the condition of the cell monolayers was verified under an inverted contrast phase microscope. The cell cultures in the medium alone were used as a positive control (PC) of cell viability (100% viable cells). To quantify the cell viability, 20 µL of MTT was added to each well, and incubation was carried out for the next 4 h. The plates were centrifuged (450× *g*, for 10 min), and the formazan crystals were dissolved with 100 µL of dimethyl sulfoxide (Sigma Aldrich, Seelze, Germany). The absorbance was measured spectrophotometrically using a Multiskan EX reader (Thermo Scientific, Waltham, MA, USA) at 570 nm.

### 4.7. Reactive Oxygen Species

Intracellular ROS were determined in AGS cells after 30 min and 24 h incubation with PyoM or LPS alone or in a combination of both stimulators, using the 2′,7′-dichlorodihydrofluorescein diacetate (H_2_DCFDA) fluorescent probe (Thermo Scientific, Waltham, MA, USA) as recommended by the manufacturer. The AGS cell suspension in the culture medium (5 × 10^5^ cells/mL) was distributed in 96-well black plates and left for 24 h (37 °C, 5% CO_2_). Afterwards, cells were treated for 30 min or 24 h with PyoM_insol_ or PyoM_sol_, in the presence or absence of *H. pylori* LPS or *E. coli* LPS, and then centrifuged (200× *g*, 5 min). After stimulation, the supernatants were replaced with 200 µL 10 µM H_2_DCFDA, and incubated for 30 min (37 °C, 5% CO_2_). Next, the cells were washed with Hanks’ Balanced Salt Solution (HBSS) and suspended in 5 mM glucose solution in HBSS (200 µL/well). Fluorescence was measured at 495 nm (excitation) and 525 nm (emission) using a SpectraMax^®^ i3x Multi-Mode Microplate Reader (Molecular Devices, San Jose, CA, USA). The ROS Index was calculated based on relative fluorescence units (RFU) of stimulated cells versus RFU of control cells in the cell culture medium alone.

### 4.8. Apoptosis

The cell apoptosis was assessed using the commercial terminal deoxynucleotidyl transferase dUTP nick end labelling (TUNEL) assay (Cell Meter TUNEL Apoptosis Assay Kit, AAT Bioques, Sunnyvale, CA, USA), as recommended by the manufacturer. Cells (5 × 10^5^ cells/mL) after stimulation for 24 h with PyoM_insol_ or PyoM_sol_ alone, with *H. pylori* LPS or *E. coli* LPS alone, were treated with fluorescent red dye that passively enters cells and selectively targets the nicks in DNA that form during apoptosis. The fluorescence of cells undergoing apoptosis was measured at 550 nm (excitation) and 590 nm (emission) using a SpectraMax^®^ i3x Multi-Mode Microplate Reader (Molecular Devices, San Jose, CA, USA). The Apoptotic Index was calculated based on the relative fluorescence units (RFU) of treated cells versus the RFU of control cells in the cell culture medium alone.

### 4.9. Wound Healing Assay

AGS cells’ migration ability was assessed in a “wound healing assay”, as previously described [16]. Cells were seeded in six-well plates at the density of 5 × 10^5^ cells per well in DMEM/F-12 medium with 2% FCS, penicillin (100 U/mL) and streptomycin (100 µg/mL), and incubated in a humidified cell incubator (37 °C, 5% CO_2_) until reaching optimal confluency. The cell monolayers were scratched with a sterile 200 µL pipette tip and designated as the start (time 0 h) of wound repair. Next, the culture medium was replaced with a solution of PyoM alone, bacterial LPS alone or both in a volume of 1 mL. Non-exposed cells in a culture medium alone were used as a control, exhibiting the spontaneous cell migration capacity. Wound images were taken at 0, 24, 48 and 72 h using a digital camera combined with an inverted contrast phase microscope (Motic AE2000, Xiamen, China), and the wound area was measured using the software Motic AE (version Motic Images Plus 2.0ML) (Xiamen, China). The wound healing in the milieu of tested formulations was expressed as the percentage of cells migrating to the wound zone compared to untreated cells.

### 4.10. Phagocytosis

The suspension of THP-1 monocytes in RPMI-1640 culture medium (5 × 10^6^ cells/mL) was applied to the wells of a 96-well plate (100 µL/well), and cells were stimulated for 24 h with PyoM_insol_ or PyoM_sol_, with or without *H. pylori* LPS or *E. coli* LPS. Before the experiment, live *H. pylori* rods were stained with the commercial LIVE/DEAD BacLight (TermoFisher, Waltham, MA, USA) for 30 min at room temperature, then added into the wells containing the monocytes at a multiplicity of infection (MOI) of 50:1 and incubated for another 30 min. Next, the cells were washed five times with 5 mM glucose solution in HBSS, and the fluorescence (excitation wavelength of 485 nm and emission wavelength of 498 nm) was measured using a multifunctional reader SpectraMax i3 (Molecular Devicesat, San Jose, CA, USA). Phagocytic activity of THP-1 cells was also assessed using the reference fluorescently labelled bioparticles, pHrodo™ Green *E. coli* BioParticles™ Conjugate for Phagocytosis Kit (ThermoFisher Scientific, Waltham, MA, USA), as recommended by the manufacturer. Fluorescent *E. coli* were suspended in HBSS supplemented with glucose to a concentration of 1 mg/mL, sonicated for 10 min and transferred to plates (100 µL/well) containing monocytes. Following the 30 min incubation, the cells were washed three times with 5 mM glucose solution in HBSS, and the intensity of fluorescence was measured using a SpectraMax^®^ i3x Multi-Mode Microplate Reader (Molecular Devices, San Jose, CA, USA) at 509 nm (excitation) and 533 nm (emission). The Phagocytic Index was calculated based on relative fluorescence units (RFU) of stimulated cells versus RFU of control cells in the cell culture medium alone.

### 4.11. Statistical Analysis

The Kolmogorov–Smirnov test was used to test the normality of the data. Intergroup outcomes were compared for statistical significance using ANOVA (analysis of variance) followed by Dunnett’s post hoc test. In all cases, significance was accepted at *p* < 0.05. All analyses were performed using GraphPad Prism 9 software (GraphPad Software, San Diego, CA, USA).

## 5. Conclusions

Our findings suggest that PyoM isolated from *Pseudomonas aeruginosa* exhibits antibacterial properties against selected reference and clinical antibiotic-resistant strains of *H. pylori*. In this study, we showed that the cytotoxicity of *H. pylori* LPS towards the reference mouse fibroblasts, human monocytes and gastric epithelial cells was reduced in the presence of PyoM. This phenomenon was related to diminished oxidative stress and apoptosis. In addition, our study also demonstrated that PyoM upregulated the phagocytosis of *H. pylori*, however, PyoM_sol_ showed better properties than PyoM_insol_ in terms of enhancing the phagocytic activity of monocytes and increasing migration of gastric epithelial cells. PyoM may represent an interesting biomolecule in further studies of the immune response modulation during *H. pylori* infection.

## Data Availability

The data generated during this study are available from the University of Łódź, Faculty of Biology and Environmental Protection, Department of Immunology and Infectious Biology, Łódź, 90-237, Poland, and are available from the corresponding authors upon request.
